# Epidemiology and survival of HPV-related tonsillar carcinoma

**DOI:** 10.1002/cam4.212

**Published:** 2014-03-10

**Authors:** Georgios Psychogios, Christoph Alexiou, Abba Agaimy, Kathrin Brunner, Michael Koch, Konstantinos Mantsopoulos, Andrea Tomppert, Heinrich Iro

**Affiliations:** 1 Department of Otorhinolaryngology, Head and Neck Surgery, Friedrich Alexander University of Erlangen-NurembergWaldstrasse 1, Erlangen, 91054, Germany; 2 Institute of Pathology, Friedrich Alexander University of Erlangen-NurembergErlangen, 91054, Germany

**Keywords:** HPV, oropharyngeal cancer, p16 oncoprotein, surgery, survival, tonsillar carcinoma

## Abstract

The aim of this study was to determine the proportion of human papilloma virus (HPV)-positive cases in tonsillar carcinomas and investigate its development over the last decade. Further aim was to show the oncologic results in accord to HPV status and various treatment modalities. A retrospective study was conducted between 2000 and 2012 and included 275 patients treated for tonsillar carcinoma. P16 immunohistochemistry was used as a surrogate marker for HPV-associated carcinogenesis. A total of 101 (36.7%) patients proved to be p16 positive and 174 p16 negative. 80.2% of the p16-positive cases presented with T1-2 tumor. Of the early-stage patients, 79% of the p16-positive and 52.3% of the p16-negative presented with lymph node metastases. The percentage of p16-positive patients increased from 23.2% in the period 2005–2007 to 58.6% in the period 2010–2012 in the whole population and from 30.9% to 76.9% in T1-2 carcinomas. Early T-category p16-positive carcinomas had significantly better disease-specific survival (92.4% vs. 75.5%, *P* = 0.007) and overall survival (OS, 79.6% vs. 54.3%, *P* < 0.001) compared to p16-negative tumors. This study showed an increase in the percentage of p16-positive patients in tonsillar carcinoma from 23.2% in the years between 2005 and 2007 to 58.6% between 2010 and 2012. The majority (80.2%) of p16-positive patients presented with early T-category tumor but most of these (79.0%) had also lymph node metastases. Nevertheless, p16-positive patients had excellent oncologic results after surgery and adjuvant radiotherapy and could be considered for de-escalation of treatment.

## Introduction

The role of human papilloma virus (HPV) in head and neck carcinoma and particularly in oropharyngeal carcinoma (OPC) has gained a great deal of attention in recent years. In contrast to other regions in head and neck oncology, the incidence of OPC has increased significantly in many countries 1. This continuous rise has been mainly attributed to HPV infection. Näsman et al. reported an increase in the proportion of HPV-related tonsillar and base of the tongue carcinomas in the Stockholm region, from approximately 23% in the 1970s to 93% in 2007 2. A recent systematic review and meta-analysis by Mehanna et al. showed that the proportion of HPV-related OPC has increased significantly worldwide from 40.5% in studies recruiting patients before 2000 to 72.2% in studies recruiting patients after 2005 3.

In addition to its causative role, HPV infection also proved to have a prognostic value in many studies. In a large, randomized, controlled study, Ang et al. showed that HPV status is an independent prognostic factor for survival in OPC 4. Other studies also demonstrated a significant survival benefit for HPV-positive OPC 5. Radiochemosensitivity of HPV-positive carcinomas has therefore been assumed and an alteration of treatment modality according to HPV status has been discussed 6. The aim of this study was to determine the proportion of HPV-positive cases in tonsillar carcinomas and to investigate its development over the last decade. A further aim was to show the oncologic results according to HPV status.

## Methods

A total of 457 patients who referred to our academic tertiary referral center (Department of Otorhinolaryngology, Head and Neck Surgery, University of Erlangen-Nuremberg Medical School, Erlangen, Germany) between 2000 and 2012 with previously untreated squamous cell carcinomas (SCC) of the tonsillar region were considered for selection. Those patients were included about whom information about HPV status could be collected. HPV infection in tumor tissue was retrospectively determined using p16 immunohistochemistry as a highly sensitive and specific surrogate marker for HPV-associated carcinogenesis 7. P16 immunohistochemistry was performed using a primary antibody retrieved from Santa Cruz Biotechnology (clone JC8, dilution: 1:100). Tumors were considered positive for p16 when strong nuclear and cytoplasmic staining was present in >60% of cells 7. The status of p16 oncoprotein expression was successfully determined in 275 patients with available paraffin blocks. In 182 cases, tumor blocks were lost or damaged. Dependencies with regard to the two groups were controlled and rated using a chi-square test and phi coefficient.

Staging was reevaluated after reviewing pretherapeutic imaging, surgical, and pathology reports according to the 2010 American Joint Committee on Cancer (AJCC) and Union Internationale Contre le Cancer (UICC) classification 8. Standard diagnostics included clinical examination, ultrasonography, and computed tomography. Magnetic resonance imaging (MRI) was also used in a few cases. The appropriate treatment modality was decided by our interdisciplinary tumor board. Factors influencing the decision were the operability of the tumor, general health status, and personal preference of each patient.

The primary endpoint of the study was to examine the development of the proportion of p16-positive patients over the last decade. Further endpoints of the analysis were disease-specific survival (DSS), local control (LC), and regional control (RC). DSS was defined using the time from the date of diagnosis to death from the cancer or complications of treatment or last follow-up. Time to LC or RC was calculated from the date of initial diagnosis to the date of most recent clinical review when local or regional recurrence was confirmed or last follow-up. Local recurrence was defined as invasive carcinoma developing after completion of initial treatment at the anatomic site of the primary tumor. Calculations of 5-year overall DSS, LC, and RC were made using Kaplan–Meier estimates and compared with the log-rank test. A *P* value of less than 0.05 was considered significant. Considering the level of measurement, multivariate analysis was performed with an appropriate logistic regression. Resultant odds ratios were rated with corresponding Wald test and Nagelkerke's pseudo-*R*^2^. All statistical analyses were performed using SPSS Version 20 (SPSS In., Chicago, IL). Relevant approval was obtained from the institutional review board of the hospital (“Ethikkommission Universität”, Erlangen).

## Results

The final study population included 275 patients who met the inclusion criteria. A total of 101 (36.7%) patients proved to be p16-positive and 174 were p16-negative. A proportion of 82.7% (225/275) of the patients presented with a primary tumor, 11.8% (32/275) with a second malignancy, and 5.5% (15/275) with multiple tumors. The median age at presentation was 57 years, ranging from 38 to 88 years (SD 9.95). The p16-positive patients had a median age of 56 years and the corresponding figure in p16-negative patients was 59 years. Fifty-three (19.3%) patients were women, with a men-to-women ratio of 4.2:1. Mean follow-up was 3.1 years (range 0.3–11.5 years). Detailed patient demographics according to p16 status are presented in Table 1.

**Table 1:** Detailed description of demographics, treatment modalities, and histological differentiation according to p16 status

Characteristics	p16-Positive (101)	p16-Negative (174)
Gender	Male: 74 (73.3%)	Male: 148 (85.1%)
	Female: 27 (26.7%)	Female: 26 (14.9%)
Age	Median: 56, range: 38–83	Median: 59, range: 42–88
Smoking	Smokers: 53 (52.5%), Ex-smokers: 15 (14.9%), Nonsmokers: 33 (32.7%)	Smokers: 121 (69.5%), Ex-smokers: 39 (22.4%), Nonsmokers: 14 (8.1%)
Primary treatment	Surgical: 86 (85.1)	Surgical: 118 (67.8%)
	Nonsurgical: 15 (14.9%)	Nonsurgical: 56 (32.2%)
Surgical technique	TLM: 11 (12.8%)	TLM: 13 (11.0%)
	Electrocautery: 71 (82.6%)	Electrocautery: 89 (75.4%)
	Combined: 4 (4.6%)	Combined: 16 (13.6%)
Adjuvant treatment	None: 15 (17.4%)	None: 35 (29.7%)
	RT: 33 (38.4%)	RT: (39.0%)
	RCT: 38 (44.2%)	RCT: 37 (31.4%)
Histological differentiation	Well-moderate (G1, G2): 51 (50.5%)	Well-moderate (G1,G2): 117 (67.2%)
	Poor – undifferentiated (G3, G4): 50 (49.5%)	Poor – undifferentiated (G3,G4): 57 (32.8%)

TLM, tranoral laser microsurgery; RT, radiotherapy; RCT, radiochemotherapy

Table 2 shows the distribution of patients according to T-category and p16 status. 80.2% of the p16-positive cases presented with early T-category. On the other hand, 50.6% of the p16-negative patients had early T-category and 47.7% had advanced T-category. Table 3 shows the distribution of patients according to N-category. Furthermore, we investigated the tendency for cervical metastases in early and advanced T-category separately, because most of the p16-positive patients presented with early T-category. Of the early-stage patients, 79% of the p16-positive, and 52.3% of the p16-negative patients presented with lymph node metastases (*P* < 0.001). Of 16 p16-positive cases that initially presented with cN0 status and underwent an elective neck dissection, 11 had at least one lymph node metastasis, giving an occult metastasis rate of 68.7% (11/16). By contrast, p16-negative patients had an occult metastasis rate of 18.2% (6/33).

**Table 2:** Number of cases according to T-category and p16 status

T-category	T1	T2	T3	T4a	T4b	Tx	All
p16 Positive	32 (31.7%)	49 (48.5%)	11 (10.9%)	7 (6.9%)	2 (2.0%)	0	101
p16 Negative	40 (23.0%)	48 (27.6%)	36 (20.7%)	42 (24.1%)	5 (2.9%)	3 (1.7%)	174
All	72 (26.2%)	97 (35.3%)	47 (17.1%)	49 (17.8%)	7 (2.5%)	3 (1.1%)	275

**Table 3:** Number of cases according to N-category, p16 status, and T subcategory

N-category		N0	N1	N2	N3	All
p16 Positive	T1-2	17 (21.0%)	17 (21.0%)	46 (56.8%)	1 (1.2%)	81
	T3-4	7 (35.0%)	0	12 (60.0%)	1 (5.0%)	20
	All	24 (23.8%)	17 (16.8%)	58 (57.4%)	2 (2.0%)	101
p16 Negative	T1-2	42 (47.7%)	12 (13.6%)	30 (34.1%)	4 (4.6%)	88
	T3-4	15 (17.4%)	3 (3.5%)	60 (69.8%)	8 (9.3%)	86
	All	57 (32.8%)	15 (8.6%)	90 (51.7%)	12 (6.9%)	174
All	81	32	148	14	275	

**Figure 1:**
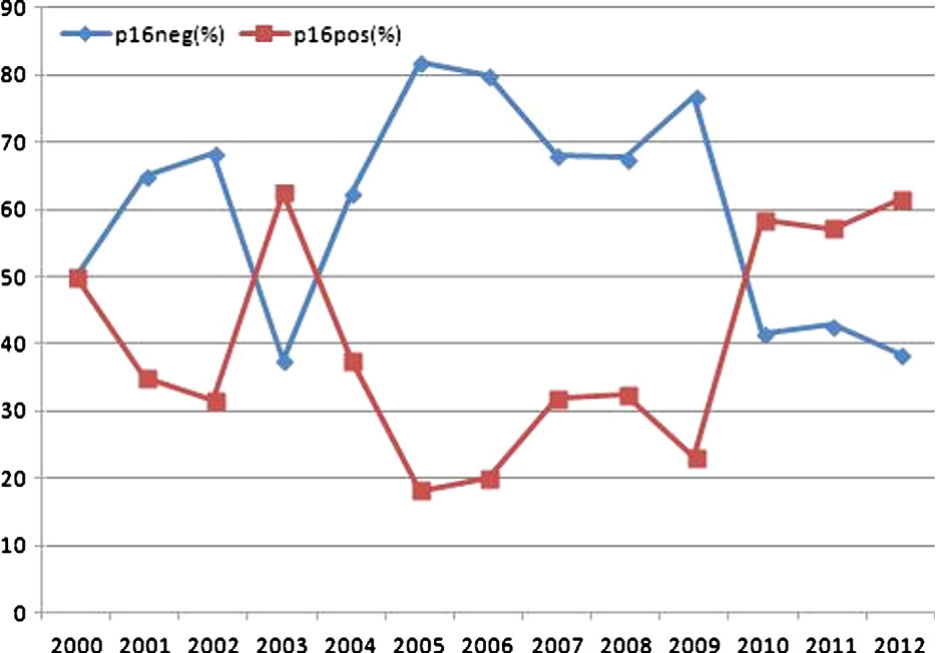
Incidence of p16-positive tonsillar carcinoma as a percentage of the whole population over the years 2000–2012

The development of incidence of p16-positive tonsillar carcinoma as a percentage of the whole population over the years 2000–2012 is shown in Figure 1. A sudden increase in the percentage of p16-positive tonsillar carcinomas can be noted from 2010 onwards. In order to improve statistical comparability of the development of incidence over the years, we divided the study period into intervals with comparable patient numbers: 2000–2004, 2005–2007, 2007–2009, and 2010–2012. The results can be seen in Table 4. The first group (2000–2004) is biased by a higher percentage of p16-positive male patients and a higher proportion of T2-carcinomas. Therefore, interval 2005–2007 was set as the reference category for further analysis. The years 2005–2007 and 2008–2009 show no significant dependence on p16 (*P* = 0.887). On the other hand, patients from the period 2010–2012 had a statistically significant higher chance of being p16-positive compared to the period 2005–2007. The increase was from 23.2% to 58.6% with Nagelkerke's pseudo-*R*^2^ of 0.099 (*P* < 0.001, OR = 4.693, Phi = 0.361, 95% CI: 2.18–10.09). If early tonsillar carcinomas (T1-2) are considered alone, then the percentage of p16-positive carcinomas also increases suddenly from 2010 onwards. The comparison of the intervals 2005–2007 and 2010–2012 revealed an increase from 30.9% to 76.9% with Nagelkerke's pseudo-*R*^2^ of 0.192 (*P* < 0.001, OR = 7.44, Phi = 0.46, 95% CI: 2.76–20.04).

**Table 4:** Development of p16-positive cases in comparison with p16-negative cases from 2000 to 2012 in accordance with T-category

Year grouped		2000–2004	2005–2007	2008–2009	2010–2012
*T1-2*	p16 Positive	25	13	13	30
	p16 Negative	19	29	21	9
All T	p16 Positive	31	16	20	34
	p16 Negative	44	53	53	24
All	275	75	69	73	58

**Figure 2:**
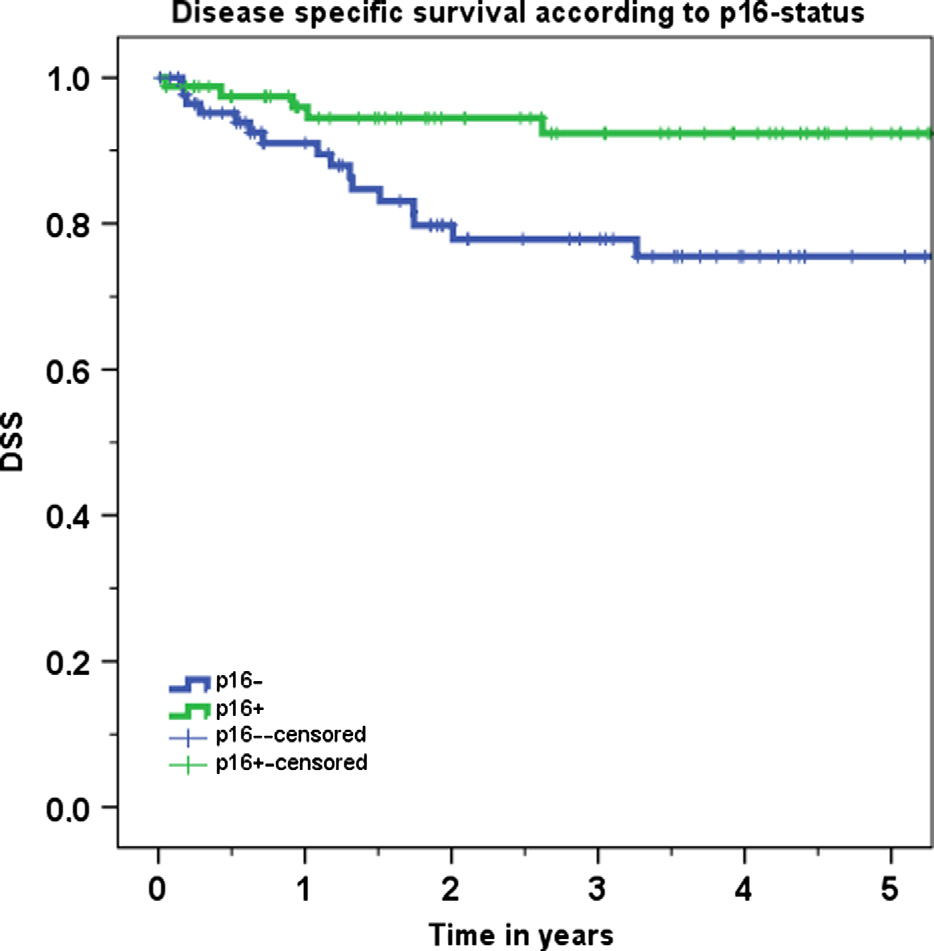
Disease-specific survival according to p16 status for T1-2 tonsillar carcinomas

The second aim of the study was to compare the oncologic results of patients with tonsillar carcinomas according to p16 status. Patients with p16-positive carcinomas (101 cases) had significantly better DSS (89.1% vs. 64.9%, *P* < 0.001) and OS (76.5% vs. 46.1%, *P* < 0.001) compared to p16-negative patients (174 cases). Due to the fact that over 80% of patients with p16-positive tumors presented with a locally early tumor (T1-2), comparison of the whole patient group is accompanied by a strong bias and therefore not acceptable. On the other hand, early T-category patients had an almost identical distribution between p16-positive (81 patients) and p16-negative (88 patients) patients. Further oncologic analysis was therefore performed in 169 patients with T1-2 tonsillar carcinoma. Primary surgical therapy was performed in 147 cases and primary nonsurgical therapy in 22 cases. Of the patients who underwent surgery, 42 had monotherapy and 113 also had adjuvant therapy. Table 5 shows the oncologic results according to p16 status in early carcinoma. Patients with p16-positive carcinomas had significantly better DSS (92.4% vs. 75.5%, *P* = 0.007) (Fig. 2) and OS (79.6% vs. 54.3%, *P* < 0.001). The results of LC and RC were flawed because of the low number of events. Although most p16-positive patients presented with lymph node metastases (64/81), they showed excellent OS (81.1%) and DSS (91.7%), as shown in Table 6. A statistical comparison was not possible because of the low number of N0 patients. On the other hand, in p16-negative patients, presence of cervical lymph node metastases had a statistically significant impact on OS (60.8% vs. 48.4%, *P* = 0.003) and DSS (82.2 vs. 69.9%, *P* = 0.041).

**Table 5:** Oncologic results according to p16 status in T1-2 tonsillar carcinoma

	5-year KM estimate (%) (Total number of events) (95% CI)				
	
	No. of patients	OS (*P* < 0.001)	DSS (*P* = 0.007)	LC	RC
p16 Positive	81	79.6 (13) (68–91)	92.4 (5) (86–99)	97.8 (1) (93–100)*	98.6 (1) (96–100)*
p16 Negative	88	54.3 (35) (42–66)	75.5 (17) (65–86)	86.6 (9) (78–95)*	87.7 (7) (79–97)*
All	169	66.0 (48) (57–75)	83.9 (22) (77–90)	92.2 (10) (87–97)	93.5 (8) (89–98)*

* No meaningful interpretation possible, due to insufficient number of events.

**Table 6:** Oncologic results according to p16 status and N-category in T1-2 tonsillar carcinoma

	5-year KM estimate (%) (Total number of events) (95% CI)			
	
	N0 versus N+	OS	DSS	RC
p16 Positive (81)	N0 (17)	73.2 (4) (46–100)*	– (1) (–;–)*	– (0) (–;–)*
	N+ (64)	81.1 (9) (68–94)	91.7 (4) (84–100)*	– (1) (–;–)*
p16 Negative (88)	N0 (42)	60.8 (15) (44–77)	82.2 (7) (69–95)*	84.3(4) (70–99)*
	N+ (46)	48.4 (20) (31–66)	69.9 (10) (54–86)	91.9 (3) (83–100)*
All	169	66.0 (48) (57–75)	83.9 (22) (77–90)	93.5 (8) (89–98)*

* No meaningful interpretation possible, due to insufficient number of events.

## Discussion

The causative and prognostic association of HPV in head and neck carcinomas and particularly OPC has focused a great deal of attention on this infection 9. In older studies, there was a clear regional difference in incidence of HPV-related OPC, but recent studies show that differences in incidence between various countries, especially the United States and Europe, seem to have disappeared 3. In many countries, including the United Kingdom, the incidence of HPV-related OPC has doubled in the last 10 years. Data from Germany are very sparse and suggest an incidence of HPV-related OPC between 20 and 40% 10,11. Even within OPC, however, tonsillar and base of the tongue carcinomas seem to represent subsites of special focus. This study, therefore, also concentrates solely on tonsillar carcinoma 11–13. The weakness of this study is its retrospective nature and the use of p16 immunohistochemistry as an indirect marker of HPV-positive tonsillar carcinoma. The combination of p16 immunohistochemistry and HPV PCR testing could lead to improved validation of HPV positivity.

This study showed an overall incidence of 36.7% for HPV-related tonsillar carcinomas. As the majority of p16-positive patients presented with early local tumor, these carcinomas represent 47.9% (81/169) of T1 and T2 tumors in this study. One possible explanation for this phenomenon could be that p16-positive patients are present at a younger age and are more differentiated, therefore seeking medical care before the local tumor reached an advanced category. Another explanation could be that the presence of cervical metastases, which occurs earlier in p16-positive patients, leads them to seek medical advice earlier and also receive a more aggressive treatment. The development of the incidence of p16-positive patients over time in the last decade supplies important information about the demographics of the disease and future expectations. In this study, the percentage of p16-positive patients varied between 23.2% in the period 2005–2007 and 58.6% in the period 2010–2012. We could not identify any graduated increase in p16-positive patients over the years, but we did observe a sudden increase during the last 3 years of the study period. The same was true when only patients with T1-2 carcinomas were considered; there was an increase from 30.9% in the period 2005–2007 to 76.9% between 2010 and 2012. The reasons for this sudden increase remain unclear and although the results are statistically significant, they have to be interpreted with caution and more study years are needed before this change can be considered definitive. Nevertheless, this high incidence of HPV-related tonsillar carcinoma, particularly in early T-category, shows that preventive and therapeutic modalities should be developed to better fit the demands of this patient group.

There are a number of studies in the recent literature that identify HPV infection as an important prognostic factor leading to favorable oncologic outcomes in OPC 4,13–15. Most of these studies focus on locally advanced OPC and use radiochemotherapy as the primary treatment option. The well-known study by Ang et al., for example, does not include T1 tumors and all patients had either stage III or IV disease 4. There are very few studies that investigate local early carcinomas and the use of surgery as treatment modality 10,16. This study is the largest to date that compares p16-positive with p16-negative patients only in cases of locally early tumor extension. A statistically highly significant difference in favor of p16-positive cases was found in both OS and DSS.

Another very interesting result of our analysis is that, in most cases, p16-positive patients presented with a locally early T-category but with lymph node metastases. Therefore, although 80.2% of p16-positive patients had an early T-category, only 21.0% (17/81) of these patients also had an early stage of disease. Perhaps this also explains the highest rate of p16-positive lymph node metastases in patients with carcinoma of unknown primary. A recent study suggests that a downregulation of E-cadherin, a cell adhesion molecule, at the primary tumor site of HPV-related tonsillar carcinomas could be related to the increased tendency toward regional metastases 17. Nevertheless, p16-positive patients had excellent oncologic results even with the combination of early T-category and the presence of lymph node metastases. Most of these cases were treated with primary surgery and postoperative radiotherapy. These results contrast with recently published data on surgically treated early OPC 10. In that study, 31 p16-positive patients with T1-2 N0-1 OPC had similar OS (80.8% vs. 79.5%, *P* = 0.59) and DSS (95.2% vs. 91.9%, *P* = 0.44) to 52 p16-negative patients. Perhaps the reason for this discrepancy was that this study excluded patients with advanced N-category (N2-3), therefore leading to excellent oncologic results even in p16-negative patients. The results of this study strengthen this hypothesis, as the presence of cervical metastases had a significant impact on survival of p16-negative (DSS 82.2 vs. 69.9%, *P* = 0.037) but not p16-positive patients. Future studies should clarify why HPV-related carcinomas so frequently lead to regional metastases and why the impact on survival in these cases is not as devastating as with lymph node metastases in all other head and neck carcinomas.

This study is the first to show that p16-positive carcinomas not only frequently present with lymph node metastases but that they also have a very high rate of occult cervical metastases (68.7%), in cases with cN0 status. This rate is not only much higher compared to p16-negative patients but also compared to advanced carcinomas in other head and neck regions 18. The logical consequence would be to perform an elective neck dissection in all patients with p16-positive tonsillar carcinomas, but as many studies failed to show a survival benefit after elective neck dissection in head and neck carcinomas 19–21, future studies should answer this question specifically for HPV-positive cases. Furthermore, a possible indication for a contralateral neck dissection should be investigated in HPV-positive patients. Although previous studies showed a very low incidence of contralateral lymph node metastases in tonsillar carcinoma, the situation might be different if only HPV-positive cases are investigated 22. The higher rate of lymph node metastases could also explain the higher risk of distant metastases and poor survival found in a recent study with advanced HPV-related OPC 23.

There has been a great deal of discussion on de-escalation of treatment in HPV-positive patients 24. The good oncologic results obtained in this patient group and the younger age at presentation make the long-term toxicity of the therapy and functional problems even more devastating for these patients. Most studies currently being performed concentrate on reducing toxicity by using induction therapy and a reduced radiation therapy 25,26. Nevertheless, local early OPC would present ideal candidates for primary surgical treatment. A transoral resection of the tumor is possible in most cases. The development of laser microsurgery and transoral robotic surgery improves the efficacy of surgical treatment 27–30. Many studies have confirmed that, with primary surgical treatment, excellent oncologic results with acceptable functional results can be achieved 31. A recent study showed that 266 patients with T1-2N0-1 OPC treated with surgery ± adjuvant therapy had DSS of 88.7% 10. In their study that included 223 patients with primary surgically treated T1 OPC, Karatzanis et al. showed that no fatal complications occurred 32. The overall complication rate was 6.2%, permanent tracheotomies were necessary in 3.1% of cases and no permanent gastrostomies were necessary. This study shows that p16-positive patients with early local tumor and presence of lymph node metastases would be ideal cases for de-escalation of treatment. Future trials should investigate whether primary transoral surgery with neck dissection as monotherapy could achieve similar oncologic results compared to those seen in patients after use of adjuvant radiotherapy. Alternatively, the effectiveness of a reduced dose of adjuvant radiotherapy could also be investigated.

Although most studies concentrate on HPV-related OPC, HPV-negative OPC should not be neglected, as they still account for the majority of patients treated 6. Most studies in the literature show poor results after combinations of radiation and chemotherapy 4. This study shows that the use of primary surgery (mostly with adjuvant radiotherapy) can lead to acceptable oncologic results in this patient group and can be used as a basis for future trials.

## Conclusion

This study showed an increase in the percentage of p16-positive patients in tonsillar carcinoma from 23.2% in the years between 2005 and 2007 to 58.6% between 2010 and 2012. The majority (80.2%) of p16-positive patients presented with early T-category tumor but most of these (79.0%) had also lymph node metastases. The p16-positive cases also had a very high rate of occult cervical metastases (68.7%). Nevertheless, p16-positive patients had excellent oncologic results and could be considered for de-escalation of treatment.
